# Improving Pregnant Women’s Iodine Intake Estimates and Its Prevalence of Inadequacy through the Use of Salt and Seasoning Covariates

**DOI:** 10.3390/nu15040846

**Published:** 2023-02-07

**Authors:** Débora L. F. Silva, Sandra P. Crispim, Claudia C. B. Almeida, Vanessa Schrubbe, Francilene M. Azevedo, Franciane R. de Faria, Nathalia Pizato, Renata J. Pereira, Mariana de S. Macedo, Sylvia do C. C. Franceschini

**Affiliations:** 1 Departamento de Nutrição e Saúde, Universidade Federal de Viçosa, Viçosa 36570-900, Minas Gerais, Brazil; 2 Departamento de Nutrição, Universidade Federal do Paraná, Curitiba 80210-170, Paraná, Brazil; 3 Faculdade de Ciências da Saúde, Universidade Federal de Rondonópolis, Rondonópolis 78736-900, Mato Grosso, Brazil; 4 Departamento de Nutrição, Universidade de Brasília, Brasília 70970-000, Distrito Federal, Brazil; 5 Curso de Graduação em Nutrição, Universidade Federal do Tocantins, Palmas 77001-090, Tocantins, Brazil; 6 Departamento de Nutrição, Universidade Federal do Vale do Jequitinhonha e Mucuri, Diamantina 39100-000, Minas Gerais, Brazil

**Keywords:** pregnant woman, food intake, diet, iodine, Brazil

## Abstract

(1) Measuring usual iodine intake is a complex task due to the food consumption variability and its natural concentration in food. Therefore, the use of covariates to adjust statistical methods to estimate usual intake could improve the estimates obtained through dietary surveys. This study aims to evaluate the influence of salt and seasoning usage covariates on the estimates of usual iodine intake and the prevalence of its inadequacy. (2) A cross-sectional study was conducted with Brazilian pregnant women’s food consumption data obtained with 24-h recall (*n* = 2247). The usual iodine intake was adjusted for intraindividual variability, supplement use, temporal effects, data collection methods, and sociodemographic characteristics with the tool UCD/NCI SIMPLE in the SAS software. Then, salt and seasoning usage covariates were used to adjust the distribution. The harmonized intake reference values for populations were used to assess intake adequacy. (3) The adjustments for salt and seasoning usage yielded a higher mean of usual iodine intakes. The only exception was the adjustment for the “habit of adding salt to meals after preparing/cooking”, which produced a lower mean of usual intake and increased the prevalence of insufficient intake. (4) Salt and seasoning usage covariates affect the estimates evaluated. However, more studies are necessary to evaluate the influence observed.

## 1. Introduction

The iodine intake of pregnant women is determined by biological, environmental, socioeconomic and political factors, making their food consumption variable and the promotion of adequate intake of this population group a challenge [1]. Considering this multifactorial influence, access to iodized salt has been critical to adequate iodine intake [2].

Iodized salt plays a key role in ensuring iodine intake as the iodine content in many foods is uncertain, particularly for that of non-marine origin. For these food items, their iodine concentration is dependent on soil type, water properties and on technologies provided by the food industry [3,4]. As a result, the variability of iodine intake is high [5,6], which makes the measurement of usual iodine intake complex. Indeed, it is estimated that 588 and 25 days of weighed food records would be necessary to obtain women’s iodine intake estimates with a 5% accuracy at the individual and population levels, respectively [6].

The interest in the usual intake occurs because it reflects the long-term intake of individuals, and it represents the most relevant exposure factor to nutritional disorders such as nutritional deficiencies [7]. Inaccurate estimates of intake have significant implications for public health, such as difficulty to identify and intervene on the population’s nutritional requirements and inefficient allocation of financial resources [8].

The 24-h recall is recognized as the best dietary method to assess and monitor the intake of individuals and population groups [9]. However, 24-h recall requires multiple applications to estimate usual intake, which increases the required human and financial resources, as well as the burden for interviewees of population studies [7,9,10]. Statistical methods to estimate usual intake adjusted for intraindividual variability were developed to overcome the limitations that are intrinsic to the multiple applications of 24-h recalls. These statistical methods require a reduced number of dietary assessments, at least one for the entire sample and a second one for a subsample. For some of these methods, covariates from food frequency or food propensity questionnaires can be added [11,12,13,14,15], which seems to improve the estimates, especially for food consumed episodically, e.g., fish [11,12,16,17,18]. Including this type of food consumption data as covariates make the symmetrical and skewed intake distribution more feasible and the intake estimates more realistic, mainly for those of the lower and upper percentiles of the distribution [12]. For nutrients consumed daily, this improvement needs further exploration [18].

To our knowledge, simulations on the performance of statistical methods to estimate usual intake adjusted for intraindividual variability were limited to individual covariates such as gender and age [19,20]. Furthermore, only two studies with pregnant women estimated the usual iodine intake and adjusted it for intraindividual variability. However, the methods applied in these studies did not allow the use of individual covariates in the adjustment of the estimates [21,22].

The monitoring of dietary intake among pregnant women allows the implementation and improvement of public health strategies needed to ensure adequate food consumption of this group [23]. However, for these strategies to be successful the intake estimates must reflect their usual intake, which is a complex measurement for iodine because of its variability in food consumption. Recognizing the complexity of the issue, we acknowledge the importance of iodized salt and seasoning in providing adequate iodine intake in areas such as Brazil where salt iodization is mandatory. Based on this understanding, we proposed that incorporating salt and seasoning as covariates in the statistical methods for usual intake assessment could improve the accuracy of iodine intake estimates obtained through dietary surveys. Therefore, this study aims to evaluate the influence of salt and seasoning usage covariates on the estimates of usual iodine intake and the prevalence of inadequate intake in Brazilian pregnant women.

## 2. Materials and Methods

### 2.1. Study Design

It is a cross-sectional study conducted with data from the Multicenter Study of Iodine Deficiency (EMDI-Brazil). The EMDI-Brazil was a national survey conducted in 11 municipalities, funded by the Ministry of Health, and designed to evaluate the iodine, sodium, and potassium nutritional status of Brazilian pregnant women, nursing mothers, and infants [24].

The EMDI-Brazil was approved (n. 2.496.986) by the Research Ethics Committees of the Federal University of Viçosa.

### 2.2. Study Location 

Brazil has continental dimensions (8,510,345.358 km^2^) and is organized into 5 macro-regions, 26 states, a Federal District, and 5570 municipalities. Its population (estimated at 213 million) [25] has an adequate nutritional status of iodine according to the median urinary iodine concentration of schoolchildren (276 µg/L) [26].

Salt Iodization was instituted as a mandatory policy in Brazil in the 1950s. Its monitoring and evaluation are part of the actions of the National Program for the Prevention and Control of Iodine Deficiency Disorders (*Pró-Iodo*) [27]. Currently, one kilogram of salt for human consumption must contain 15–45 mg of iodine, and industrialized food is exempted from adding iodized salt if duly proven that such addition causes organoleptic changes in the product [28]. It is also worth mentioning that *Pró-Iodo* does not require Brazilians to supplement iodine.

### 2.3. Study Population

This study included pregnant women over 18 years old, users of the National Health System (hereafter SUS) with plausible food consumption. Pregnant women with thyroid diseases (hypothyroidism, hyperthyroidism, Hashimoto’s disease, and neoplasms) or thyroid gland surgery were not included [24].

The sampling strategy was based on EMDI-Brazil’s predefined parameters: minimum expected iodine deficiency prevalence of 8% with a relative error of 50% (range from 4% to 12%) and a confidence level of 95%. These parameters resulted in a simple random sample of 177 individuals per municipality. Given that this is a complex sample selected from the units of the Family Health Strategy that make up the primary health care network of each municipality, the effect of the sample plan (design effect) of 1.5 was included, which increased the sample size to 266 pregnant women in each collection center [24].

The pregnant women were selected from a stratified sampling plan with a two-stage draw [29]. The municipalities’ basic health units (hereafter UBSs) were the primary sampling unit, and the pregnant women attending these UBSs were the secondary ones. Due to logistical difficulties, partly due to the COVID-19 pandemic, pregnant women were approached while visiting the UBSs during their monthly pregnancy monitoring and invited to participate in the study voluntarily. Those who accepted the invitation read and signed the Informed Consent Form.

### 2.4. Data Collection

The data were collected between September 2018 and April 2021. The EMDI-Brazil Coordination previously trained the field teams to conduct face-to-face interviews at the UBSs or, in a few cases, during a home visit [24].

Pregnant women’s socioeconomic, demographic, and lifestyle data were collected through a structured questionnaire incorporated in the REDCap (Research Electronic Data Capture) [30]. Salt from the households of a subsample of the pregnant women and drinking water from the UBSs were collected for iodine concentrations assessment through the methodology defined by EMDI-Brazil [24]. The iodine concentration in the salt was measured using an analytical method in which potassium iodate of the salt reacts in an acidic solution releasing iodine, and is later titrated with sodium thiosulfate [31]. The iodine concentration in drinking water was quantified by the spectrophotometric method based on leuco crystal violet, which determines aqueous iodine in the form of elemental iodine and hypoiodous acid [32].

The use of supplements, as well as their information on the type, brand, and dosage, were investigated during the application of the structured questionnaire.

### 2.5. Dietary Intake Data 

Food consumption data were collected in interviews conducted by the Multiple Pass Method (MPM) [33] using a paper-based 24-h recall (available at: www.gupea.ufpr.br (accessed on 20 April 2019)) and the “Brazilian Manual for Food Portion Quantification” [34]. One 24-h recall was applied in the entire sample and a second one in a subsample with a minimum interval of one week. These recalls were collected on different days of the week, 81.7% referring to food consumption from Monday to Thursday, as well as in different seasons (24.3% spring; 25.3% summer; 28.0% autumn; 22.4% winter).

Food consumption data were then entered in the Data Entry application of GloboDiet’s Brazilian version [35]. After that, data quality control took place. Inconsistencies in the description or quantification of food consumption were treated in a standardized way as per the guidelines in the “Standardization Manual of the Treatment of Notes in GloboDiet” developed by the research group.

The food consumed were correlated with information from the Food Iodine Content Table (FICT) [36], the Brazilian Food Composition Table (hereafter TBCA) [37], and from food labels at the level of food and ingredients recipes [38]. EMDI-Brazil researchers developed FICT to meet the need for information about iodine concentration in foods consumed by Brazilians. When a food iodine concentration was not found in the FICT, other food composition tables were consulted by a pair of researchers [39]. In cases where the composition of a food item could not be identified from these tables, it was treated as a missing value, which affected 2.8% of food items [39]. For salt and water iodine concentration, we used the median iodine concentration from the biochemical analysis performed by EMDI-Brazil [24,39]. Later, the iodine concentration of the dietary component “addition salt (g)” from TBCA was incorporated into the food composition database as additional rows for each pregnant woman.

24-h recalls with energy intake from 500 Kcal/day to 4000 Kcal/day [7] and with at least five food items were included in the analyses. For recalls that did not meet these criteria, a biological plausibility criterion was used to decide for its inclusion. This criterion included reports of nausea, vomiting, and excessive appetite or increased consumption due to an atypical day. At the end, 52 24-h recalls were considered implausible, and data from 2247 pregnant women were used in this analysis. Replication was possible in 18.3% of these recalls.

### 2.6. Data Analysis

The Macro UCD/NCI Simulating Intake of Micronutrients for Policy Learning and Engagement (SIMPLE) was used to estimate the usual iodine intake adjusted for intra-individual variability [13]. The UCD/NCI SIMPLE combines the macros of the National Cancer Institute (NCI) method to estimate usual intake distributions of food and nutrients consumed “nearly daily”, and the prevalence of inadequate intake (insufficient and excessive). In addition, this macro allows the inclusion of different covariates to adjust the usual intake estimates [13], such as supplement use, temporal effect, method used to collect dietary data, sampling, and individual characteristics. The SIMPLE macro was used to estimate iodine and salt intake of the sample.

In our analyses, we proposed different models to adjust the usual iodine intake of pregnant women (Table 1). These models were based on relations identified in studies that quantified and evaluated iodine intake or iodine nutritional status of pregnant women, and also on variables of interest related to Nutritional Epidemiology [1,40,41,42,43,44,45,46,47,48,49,50,51,52].

In the Baseline Model, the usual iodine intake from food consumption was adjusted for intraindividual variability. Iodine from “iodine-containing supplements use” (user or non-user; and amount used (g)) was then incorporated into the adjustment (Model 1), followed by the temporal effects and method used to collect dietary data (“recalled day of the week” (weekday (Monday to Thursday), weekend (Friday to Sunday), “season” (spring; summer; autumn; winter) and “24-h recall sequence” (1st or 2nd)) (Model 2). Afterward, from Model 3, individual covariates (“municipality location” (coastal; countryside); “age” (in years), “color/race” (white and yellow; black, parda (mixed) and indigenous), “schooling” (no education and elementary school; high school; graduate or postgraduate) and “gestational age” (first, second or third trimester)) were used to adjust the models.

The salt and seasoning covariates were used in Models 4 to 34 by incorporating “type of salt used “(none; refined iodized salt; others); “use of pure salt” (yes; no); “habit of adding salt to meals after preparing/cooking” (yes; no); “use of homemade seasoning” (yes; no); and “use of industrialized seasoning” (yes; no) information. From the fourth to the eighth model, salt and seasoning usage covariates were used to adjust each model individually to observe its isolated effect on the estimates of usual intake and prevalence of inadequate intake. Finally, the combined effect of these covariates on the estimates was evaluated from Model 9. Because the level of importance of the salt and seasoning covariates on these estimates was unknown, we chose to test different combinations of these covariates. The order of entry of the combined covariates in the models were made automatically by the UCD/NCI SIMPLE’s statistical procedures.

The iodine intake harmonized reference values (H—AR (160 mcg) and H—UL (600 mcg)) were used to assess the prevalence of insufficient and excessive intake, respectively. These values were used because they can be considered an international dietary intake reference for the assessment, planning, and comparison of pregnant women’s iodine intake [53].

Pregnant women’s characteristics and iodine intake were described by the mean, 95% confidence interval (95% CI), percentiles and/or frequencies (absolute and relative). Other results were expressed by the prevalence of inadequate intake (insufficiency and excess) and by the parameters of the intake distribution (Akaike Information Criterion (AIC), lambda, variances (total variance (S^2^), intraindividual variability (S^2^w) and interindividual variability (S^2^b)), and ratio of intraindividual to interindividual variability (S^2^w/S^2^b)).

The coefficient of variation (CV) and skewness of iodine intake distributions from the models were quantified and compared as metrics of relative variability and asymmetry of these distributions.

All categorical variables were converted into dummy ones to adjust the models. Finally, the results were generated by the SIMPLE macro except for individual characteristics, CV, and skewness.

The AIC was used to identify the best model for adjusting the usual iodine intake. By this criterion, the lower the value yielded by a model, the greater its ability to explain data with fewer parameters adequately [54].

The Statistical Analysis System (SAS) software (OnDemand for academics version, SAS Institute Inc.: Cary, NC, USA) was used in the analyses [55]. It was assumed that the estimates and parameters showed a statistically significant difference when the 95% CI did not overlap.

## 3. Results

The pregnant women (*n* = 2247) had a mean age of 26.7 years (95% CI: 26.5–27.0) and were mostly countryside residents (63.6%; 95% CI:61.5–65.5), black, parda (mixed) and indigenous color/race (73.0%; 95% CI: 71.0–74.9), with high school education (62.2%; 95% CI: 60.1–64.3), in the second gestational trimester (39.7%; 95% CI: 37.3–41.8), and non-users of iodine-containing supplements (91.6%; 95% CI: 90.4–92.7) (Table 2).

Around 79% (95% CI: 76.9–80.5) of the pregnant women reported using salt in its pure form, of which 93% (95% CI: 91.8–94.0) reported the use of refined iodized salt and only 0.5% (95% CI: 0.3–1.0) reported no use of salt (Table 1). Additionally, the habit of adding salt to meals after preparing/cooking was reported by 16.1% (95% CI: 14.3–17.9) of the sample, and the mean of usual salt intake was 2.3 g (95% CI: 2.2–2.3). Regarding seasoning, 35% (95% CI: 33.0–37.1) used homemade seasoning, and 59.5% (95% CI: 57.4–61.6) the industrialized type (Table 2).

The models provided mean estimates of usual iodine intake between 136.6 and 183.8 mcg/day (Table 3) and prevalence of insufficient intake between 44.7 and 60.9%. The contribution of iodine from supplements (Model 1) was 19.8 mcg (95% CI: 19.0–20.5 mcg), which increased the mean intake of pregnant women to 182.8 mcg (95% CI: 182.0–183.6) and reduced the prevalence of insufficient intake to 45.5% (95% CI: 45.3–45.7) when compared to the Baseline Model (163.1 mcg; 95% CI: 162.9–163.2 and 49.8%; 95% CI: 49.6–50.0). The adjustment for the method used to collect dietary data and temporal effects (Model 2) did not significantly change the estimates yielded by the use of iodine-containing supplements.

The adjustment for sociodemographic characteristics (Model 3) reduced the iodine intake of the group (171.7 mcg; 95% CI: 170.9–172.5). This effect was most evident in the fifth percentile of the intake distribution, and it significantly increased the prevalence of insufficient intake (49.4%; 95% CI: 49.2–49.6) (Table 3). Nevertheless, the estimates of usual iodine intake and prevalence of insufficient intake yielded by Model 3 were better than those of the Baseline Model. Furthermore, percentages of excessive intake were only observed after the inclusion of iodine supplement usage in the models, with no difference between the excessive percentages of the models (Table 3).

The usual iodine intake of the models adjusted for salt and seasoning covariates were significantly different from that of the Baseline Model. The magnitude of these differences was dependent on the covariates incorporated in the adjustment (Table 3). The means of usual intake from these models were lower than that of the Baseline Model only when the covariate “habit of adding salt to meals after preparing/cooking” was incorporated into the adjustments. Models that did not have this covariate had higher means of usual intakes than that of the Baseline Model, but their prevalence of insufficient intake were similar.

No differences were found among models 4 to 34 other than those produced by “habit of adding salt to meals after preparing/cooking” covariate. The adjustment for this variable alone or together with other covariates caused a left deviation of the distribution, which reduced the estimates of iodine intake, mainly those of the lower percentiles (P5 and P25) (Table 3). As a result, the prevalence of insufficient intake increased by an average of 10.9% (10.7–11.1%) compared to the Baseline Model. The effect of the adjustment for the “habit of salt addition after preparing/cooking” was also observed by the reduction of the Coefficient of variation (CV) and by the change in the skewness of the distributions from positive to negative in the models (Table 4).

The model with the best fit (model 21; Akaike Information Criterium (AIC) = 21771.5) (Table 4) was adjusted for “iodine-containing supplements use,” “temporal effects”, “method used to collect dietary data”, “sociodemographic characteristics”, and then for “type of salt used”, “habit of adding salt to meals after preparing/cooking” and “use of homemade or industrialized seasoning” (Table 1). From this model, the mean of usual iodine intake was 136.6 mcg (95 CI%: 135.8–137.4 mcg), and the prevalence of insufficient and excessive intake were 60.9% (95% CI%: 60.7–61.1) and 0.1% (95 CI%: 0.1–0.1), respectively (Table 3).

All models’ variances remained similar according to the 95% CI, and intraindividual to interindividual variability ratios were between 2.3 and 3 (Table 4).

## 4. Discussion

We evaluated the effects of salt and seasoning covariates on estimates of usual iodine intake and the prevalence of inadequate intake in Brazilian pregnant women. We observed that the Models produced intake estimates different from those of the Baseline Model, which iodine intake from food was only adjusted for intraindividual variability. The adjustment for the “habit of adding salt to meals after preparing/cooking” caused a shift in intake distribution to the left, reducing the estimates of this intake and increasing the prevalence of insufficient intake. The adjustment for the remaining salt and seasoning covariates produced higher intake means compared to the Baseline Model. From the best model, the usual iodine intake mean of pregnant women was 136.6 mcg, with 60.9% and 0.1% of them having insufficient (<160 mcg/day) and excessive (>600 mcg/day) intake, respectively.

The iodized salt is the primary source of iodine in Brazil [27], which explains the close relationship between iodine intake and the amount of salt used for food preparation, cooking, and consumption [56]. Exploratory analysis of data (results not presented on tables) showed that pregnant women who reported having the “habit of adding salt to meals after preparing/cooking” also had iodine intake (164.1 mcg; 95% CI: 163.8–164.5) slightly higher than those of pregnant women who reported not having this habit (162.5 mcg; 95% CI: 162.3–162.7). However, because of the low percentage of the sample that reported having this habit (16.5%), the use and maintenance of this covariate has likely reduced the mean of usual iodine intake and increased the prevalence of insufficient intake.

The positive effect of the habit of adding salt to meals on the iodine nutritional status of pregnant women was also evidenced in a study conducted by Kasap et al. [51]. In this study 39% of the women evaluated (*n* = 135) reported adding salt to food after cooking. This habit was associated with their better iodine nutritional status [51], which seems to be related to the lower volatility of iodine in salt when it is added to food after cooking [57]. In addition, the authors highlighted the need to store iodized salt in locations and containers that reduce sunlight exposure to preserve the salt iodine content [51].

While the consumption of iodized salt is crucial for maintaining proper iodine nutrition [2], experts are now increasingly suggesting reducing salt intake to lower excessive sodium consumption and decrease the risk of cardiovascular disease. The World Health Organization (WHO) has set a global target of a 30% reduction in salt intake by 2025 [58], and the effects of this strategy on iodine intake have raised concern among countries. It raises concern because it could cause the resurgence of iodine disorders even in areas where salt iodization is mandatory and has high coverage [59]. Even so, the WHO states that the policies for salt iodization and reduction of its intake are compatible, cost-effective, and of great value to the population. According to this organization, the concentration of iodine in salt can be adjusted as salt intake is reduced (5 g/day of salt with 50 mg/Kg of iodine, for example) [59]; however, countries have shown difficulty to manage this recommendation [2].

Since the reduction of salt iodine concentration in Brazil in 2013 [28], the assessment of iodine intake of its pregnant women had not been performed. According to the country’s Health Authorities, this reduction was needed due to excessive salt intake by the Brazilian population (around 12 g/day), estimated from sodium intake (4.7 g/day) available in the *Pesquisa de Orçamentos Familiares* (POF—Household Budget Survey) (2008–2009) [60]. In our study, however, we observed that the salt intake of the surveyed pregnant women was lower (2.3 g) than that highlighted in 2008, and also lower than the 9.35 g/day identified for Brazilians at the *Pesquisa Nacional de Saúde* (PNS—Bazilian Health Survey) in 2013 [61].

The lower salt intake observed in this study may be overlooked as a positive factor to prevent cardiovascular diseases, but it can represent a risk of insufficient iodine intake. We must also point out that this salt intake may have been a consequence of the lower capacity of dietary assessment methods to measure this food item when compared to 24-h urinary sodium excretion method, considered the gold standard for this quantification [61,62]. This limitation was also highlighted by Sarno et al. [60] in the assessment of sodium intake using data from the “individual consumption” module in the POF (2008–2009).

The quantification of salt and, consequently, of its minerals, should be considered a limitation of food consumption studies given the variation of salt usage in homemade preparations, industrialized foods, and saltshakers [56,57,63]. To overcome this limitation, urinary iodine concentration is usually used as the indicator of recent iodine intake, but its operationalization and cost limit its use in population studies [64,65]. Hence, for accurate estimates of iodine intake by dietary surveys, special attention is required during the collection of salt usage data. This attention concerns mainly the type and quantity of salt used in food preparation, cooking, and consumption, as well as on the brands of industrialized food, especially in areas where salt iodization legislation is flexible for these type of products [63], such as Brazil [28].

As mentioned, not all industrialized Brazilian foods must contain iodized salt in their composition [28]. Research conducted by the authors on the country’s three leading brands of seasoning showed that the labels of their products have no mention or indication that they had been manufactured with iodized salt. Moreover, we observed the existence of “zero salt” advertisements for some seasoning products, which do not clarify whether the iodine content was reduced or removed.

The fieldwork teams of Multicenter Study of Iodine Deficiency (EMDI-Brazil) were trained to overcome the challenges of iodine measurement during the 24-h recall interview conducted using the Multiple Pass Method (MPM) [33]. The interviewers were instructed to investigate the ingredients of the recipes: use and amount of salt added to the meals during and after the preparation or cooking process; name, brand, and the level of food processing in an attempt to measure the consumption of salt and seasoning in the best possible way. Furthermore, during data analysis, we were also attentive to adding the iodine concentration from the dietary component “addition salt (g)” of the Brazilian Food Composition Table (hereafter TBCA) [37] to the iodine intake of each pregnant woman. However, it is possible that our data collection method was not sensitive enough to accurately capture the actual consumption of these food items. It is also possible that the iodine concentration from the food composition tables were not appropriate to observe the expected effect in the proposed analyzes. Nevertheless, we recognize the efforts made by the EMDI-Brazil team to show the first evidence of iodine intake of Brazilian pregnant women and highlight the need for information about the iodine content of food items produced and consumed by people from different regions of this country.

Based on the best fit model, we observed that 61% of pregnant women had inadequate iodine intake, with the majority (60.9%) consuming less than the recommended intake. In addition, albeit reduced (0.1%), the prevalence of excessive intake related to the use of iodine-containing supplements highlights the need for regulation and monitoring of the use of these compounds by pregnant women in Brazil; it also highlights the risk of clinical or subclinical hypo/hyperthyroidism, thyroid nodules, thyroid cancer, graves’ disease, Hashimoto’s thyroiditis, and fetal hypothyroidism in this population group [66]. Conversely, these results must be interpreted with caution. Despite our efforts to improve iodine intake estimates from a dietary survey, the WHO recommend urinary iodine as the gold standard for iodine nutrition evaluation at individual and population levels given that up to 90% of dietary iodine is excreted in the urine when intake is sufficient [64,65]. Therefore, future studies should explore food consumption data with urinary iodine information.

The results of our study reinforce the need to improve the strategies of the National Program for the Prevention and Control of Iodine Deficiency Disorders (*Pró-Iodo*) [27] related to the physiological needs of pregnant women. It also demonstrates the need for the continuous assessment of iodine intake, for the appropriate use of iodine-containing supplements, and for the development and application of appropriate methodologies to measure salt and seasoning intake. In 2016, an Iodine Global Network spokesman pointed out some of these demands to the Commission of *Pró-Iodo* [67]. Unfortunately, Brazilian strategies have been restricted to monitoring salt iodine concentration at the industrial and commercial levels.

This study has limitations. First, we highlight the underestimation of food consumption given that the ratio between reported energy consumption and basal metabolism rate (1.07) was below the estimated confidence interval (1.38–1.42) [68]. However, every evaluation method has measurement errors, and underestimation is expected with the application of 24-h recalls [7]. Despite this, the conduction of the 24-h recall interview by the MPM method [33], the use of a standardized 24-h recall [35], and the use of a manual for food quantification [34] may have helped to minimize this limitation. Second, for the quantification of salt intake by pregnant women, we did not distinguish the type of salt used by them. Still, iodized salt is present in 98% of Brazilian households [2] and only 6.5% of our sample reported using salt different from the refined and iodized one. Third, the bias related to iodine concentration in foods due to its high variability and the use of information from food composition tables of other countries. Fourth, the adoption of current iodine-containing supplements as a proxy for their usual usage. Nonetheless, 71.5% of the Brazilian pregnant women underwent prenatal monitoring in SUS in 2019 [69], and our sampling power to estimate their iodine intake was 100%. This post-calculation power was based on an expected mean and standard deviation of 153.11 ± 28.83 mcg [70], with a significance level of 5% [71].

As strengths of this study, we emphasize that this is the first study to test the usage of individual covariates other than gender and age to estimate usual intake and prevalence of inadequate intake of a dietary component consumed daily by pregnant women. In addition, we would like to highlight the use of the Macro UCD/NCI Simulating Intake of Micronutrients for Policy Learning and Engagement (SIMPLE) [13] as a potential tool to optimize the use and application of food consumption data by public policymakers and researchers. Our study’s exploratory analyses based on iodine intake from food showed only slight differences between SIMPLE macro and the National Cancer Institute (NCI) macros (SAS macros version 2.1). For the estimates of usual iodine intake, this difference was a maximum of 0.2 mcg, and for the prevalence of inadequate intake, of 0.2%. Furthermore, the results of this study can be helpful to national policy makers and researchers interested in an iodine intake assessment and its relationship with the consumption of important food sources, such as iodized salt and seasoning.

To finish, our results reflect a scenario prior to the COVID-19 pandemic. Nevertheless, it constitutes evidence of how covariates related to dietary practices can influence estimates of usual intake and prevalence of inadequate intake of nutrients consumed daily.

## 5. Conclusions

We identified that covariates related to the use of salt and seasoning do influence the estimates of usual iodine intake and the prevalence of its insufficient intake, especially when they are adjusted for “habit of adding salt to meals after preparing/cooking”. However, we recommend conducting studies in areas with diverse habits of salt and seasoning usage to explore the results further.

## Figures and Tables

**Table 1:** Adjustment models to estimate the usual iodine intake of pregnant women and the prevalence of inadequate intake, EMDI—Brazil, Brazil, 2023.

Models	
Baseline	Iodine Intake from Food Adjusted for Intraindividual Variability
1	Iodine intake from food and iodine-containing supplements adjusted for intraindividual variability
2	Model 1 + recalled day of the week + season + 24-h recall sequence
3	Model 2 + municipality location + age + color/race + schooling + gestational age
4	Model 3 + type of salt used
5	Model 3 + use of pure salt
6	Model 3 + habit of adding salt after preparing/cooking
7	Model 3 + use of homemade seasoning
8	Model 3 + use of industrialized seasoning
9	Model 3 + type of salt used + use of pure salt
10	Model 3 + type of salt used + habit of adding salt after preparing/cooking
11	Model 3 + type of salt used + use of homemade seasoning
12	Model 3 + type of salt used + use of industrialized seasoning
13	Model 3 + type of salt used + use of pure salt + habit of adding salt after preparing/cooking
14	Model 3 + type of salt used + use of pure salt + use of homemade seasoning
15	Model 3 + type of salt used + use of pure salt + use of industrialized seasoning
16	Model 3 + type of salt used + use of pure salt + habit of adding salt to meals after preparing/cooking + use of homemade seasoning
17	Model 3 + type of salt used+ use of pure salt + habit of adding salt to meals after preparing/cooking + use of industrialized seasoning
18	Model 3 + type of salt used + use of pure salt + use of homemade seasoning + use of industrialized seasoning
19	Model 3 + type of salt used + habit of adding salt to meals after preparing/cooking + use of homemade seasoning
20	Model 3 + type of salt used + habit of adding salt to meals after preparing/cooking + use of industrialized seasoning
21	Model 3 + type of salt used + habit of adding salt to meals after preparing/cooking + use of homemade seasoning + use of industrialized seasoning
22	Model 3 + type of salt used + use of homemade seasoning + use of industrialized seasoning
23	Model 3 + use of pure salt + habit of adding salt to meals after preparing/cooking
24	Model 3 + use of pure salt + habit of adding salt to meals after preparing/cooking + use of homemade seasoning
25	Model 3 + use of pure salt + habit of adding salt to meals after preparing/cooking + use of industrialized seasoning
26	Model 3 + use of pure salt + habit of adding salt to meals after preparing/cooking + use of homemade seasoning + use of industrialized seasoning
27	Model 3 + use of pure salt + use of homemade seasoning
28	Model 3 + use of pure salt + use of industrialized seasoning
29	Model 3 + use of pure salt + use of homemade seasoning + use of industrialized seasoning
30	Model 3 + habit of adding salt to meals after preparing/cooking + use of homemade seasoning
31	Model 3 + habit of adding salt to meals after preparing/cooking + use of industrialized seasoning
32	Model 3 + habit of adding salt to meals after preparing/cooking + use of homemade seasoning + use of industrialized seasoning
33	Model 3 + use of homemade seasoning + use of industrialized seasoning
34	Model 3 + type of salt used + use of pure salt + habit of adding salt to meals after preparing/cooking + use of homemade seasoning + use of industrialized seasoning

**Table 2:** Characteristics of pregnant women from Multicenter Study of Iodine Deficiency, Brazil, 2023.

Characteristics	Mean		95% CI
Age (in years) (*n* = 2247)	26.7		26.5–27.0
Usual salt intake (in grams) (*n* = 2247)	2.3		2.2–2.3
	*n*	%	95% CI
Season (*n* = 2247)			
Summer	568	25.3	23.5–27.1
Autumn	629	28.0	26.1–29.9
Winter	503	22.4	20.7–24.2
Spring	547	24.3	22.6–26.2
Municipality location (*n* = 2247)			
Coastal	819	36.4	34.5–38.5
Countryside	1428	63.6	61.5–65.5
Gestational age (in trimester) (*n* = 2234)			
1	473	21.2	19.5–22.9
2	887	39.7	37.3–41.8
3	874	39.1	37.1–41.2
Color/race (*n* = 2101)			
Black, parda (mixed) and indigenous	1533	73	71.0–74.9
White and yellow	568	27.0	25.1–29.0
Schooling (*n* = 2091)			
No education and elementary school	455	21.7	20.0–23.6
High school	1300	62.2	60.1–64.3
Graduate or postgraduate	336	16.1	14.5–17.7
Use of iodine-containing supplements (*n* = 2247)			
Yes	189	8.4	7.3–9.6
No	2058	91.6	90.4–92.7
Type of salt used (*n* = 2105)			
None	12	0.5	0.3–1.0
Refined iodized	1957	93.0	91.8–94.0
Others	136	6.5	5.4–7.6
Use of pure salt (*n* = 2106)			
Yes	1658	78.7	76.9–80.5
No	448	21.3	19.5–23.1
Habit of adding salt to meals after preparing/cooking (*n* = 1632)			
Yes	262	16.1	14.3–17.9
No	1370	83.9	82.1–85.7
Use of homemade seasoning(*n* = 2098)			
Yes	735	35.0	33.0–37.1
No	1363	65.0	62.9–67.0
Use of industrialized seasoning (*n* = 2103)			
Yes	1252	59.5	57.4–61.6
No	851	40.5	38.4–42.6

Note: 95% CI. 95% confidence interval.

**Table 3:** Usual iodine intake and prevalence of inadequate intake of pregnant women according to Models, Multicenter Study of Iodine Deficiency, Brazil, 2023.

Models	Mean	95% CI Mean	Supplement Mean	95% CI Supplement Mean	P5	P25	P50	P75	P95	Ins. (%)	95% CI Ins.	Exc. (%)	95% CI Exc.
Baseline	163.1	162.9–163.2	0.0		105.0	135.8	160.2	187.2	230.7	49.8	49.6–50.0	0.0	
1	182.8	182.0–183.6	19.8	19.0–20.5	106.3	138.3	164.7	197.5	307.9	45.5	45.3–45.7	0.1	0.1–0.1
2	183.8	183.0–184.6	19.8	19.0–20.5	105.5	138.4	165.6	199.6	309.1	44.7	44.5–44.9	0.1	0.1–0.1
3	171.7	170.9–172.5	19.8	19.0–20.5	1.2	131.3	160.7	195.2	304.7	49.4	49.2–49.6	0.1	0.1–0.1
4	171.6	170.7–172.4	19.8	19.0–20.5	1.2	131.2	160.6	195.3	304.8	49.5	49.2–49.7	0.1	0.1–0.1
5	171.6	170.8–172.4	19.8	19.0–20.5	1.2	131.3	160.7	195.2	304.7	49.4	49.2–49.6	0.1	0.1–0.1
6	137.5	136.6–138.3	19.8	19.0–20.5	1.2	1.2	145.8	185.6	284.2	60.6	60.4–60.8	0.1	0.1–0.1
7	171.1	170.3–171.9	19.8	19.0–20.5	1.2	131.1	160.6	195.1	304.9	49.5	49.3–49.7	0.1	0.1–0.1
8	171.4	170.6–172.2	19.8	19.0–20.5	1.2	130.9	160.6	195.4	304.6	49.5	49.3–49.7	0.1	0.1–0.1
9	171.5	170.7–172.3	19.8	19.0–20.5	1.2	131.2	160.6	195.3	304.7	49.5	49.3–49.7	0.1	0.1–0.1
10	137.5	136.6–138.3	19.8	19.0–20.5	1.2	1.2	145.9	185.6	284.1	60.5	60.3–60.7	0.1	0.1–0.1
11	171.0	170.1–171.8	19.8	19.0–20.5	1.2	130.9	160.5	195.1	304.9	49.6	49.4–49.8	0.1	0.1–0.1
12	171.3	170.5–172.1	19.8	19.0–20.5	1.2	130.8	160.6	195.5	304.7	49.5	49.3–49.7	0.1	0.1–0.1
13	137.5	136.7–138.3	19.8	19.0–20.5	1.2	1.2	145.9	185.7	284.2	60.5	60.3–60.7	0.1	0.1–0.1
14	170.9	170.1–171.7	19.8	19.0–20.5	1.2	130.8	160.5	195.1	304.9	49.6	49.4–49.8	0.1	0.1–0.1
15	171.2	170.4–172.0	19.8	19.0–20.5	1.2	130.8	160.5	195.3	304.6	49.6	49.4–49.8	0.1	0.1–0.1
16	136.8	135.9–137.6	19.8	19.0–20.5	1.2	1.2	145.3	185.1	284.1	60.8	60.6–61.0	0.1	0.1–0.1
17	137.3	136.5–138.1	19.8	19.0–20.5	1.2	1.2	145.6	185.6	284.1	60.6	60.4–60.8	0.1	0.1–0.1
18	170.6	169.8–171.4	19.8	19.0–20.5	1.2	130.4	160.3	195.2	304.8	49.7	49.5–49.9	0.1	0.1–0.1
19	136.8	136.0–137.7	19.8	19.0–20.5	1.2	1.2	145.2	185.5	284.2	60.8	60.6–61.0	0.1	0.1–0.1
20	137.3	136.5–138.2	19.8	19.0–20.5	1.2	1.2	145.7	185.6	284.0	60.6	60.4–60.8	0.1	0.1–0.1
21	136.6	135.8–137.4	19.8	19.0–20.5	1.2	1.2	145.0	185.2	283.8	60.9	60.7–61.1	0.1	0.1–0.1
22	170.7	169.9–171.5	19.8	19.0–20.5	1.2	130.5	160.4	195.2	304.9	49.7	49.4–49.9	0.1	0.1–0.1
23	137.5	136.7–138.4	19.8	19.0–20.5	1.2	1.2	145.9	185.6	284.3	60.5	60.3–60.7	0.1	0.1–0.1
24	136.8	136.0–137.7	19.8	19.0–20.5	1.2	1.2	145.3	185.2	284.1	60.8	60.6–61.0	0.1	0.1–0.1
25	137.4	136.5–138.2	19.8	19.0–20.5	1.2	1.2	145.6	185.6	284.0	60.6	60.4–60.8	0.1	0.1–0.1
26	136.7	135.8–137.5	19.8	19.0–20.5	1.2	1.2	145.1	185.2	283.9	60.9	60.7–61.1	0.1	0.1–0.1
27	171.0	170.2–171.8	19.8	19.0–20.5	1.2	130.9	160.5	195.2	304.8	49.6	49.4–49.8	0.1	0.1–0.1
28	171.3	170.5–172.1	19.8	19.0–20.5	1.2	131.0	160.6	195.3	304.7	49.5	49.3–49.7	0.1	0.1–0.1
29	170.7	169.9–171.5	19.8	19.0–20.5	1.2	130.6	160.4	195.1	304.8	49.7	49.5–49.9	0.1	0.1–0.1
30	136.8	136.0–137.7	19.8	19.0–20.5	1.2	1.2	145.3	185.2	284.1	60.8	60.6–61.0	0.1	0.1–0.1
31	137.4	136.5–138.2	19.8	19.0–20.5	1.2	1.2	145.7	185.6	284.0	60.6	60.4–60.8	0.1	0.1–0.1
32	136.7	135.9–137.5	19.8	19.0–20.5	1.2	1.2	145.1	185.2	283.8	60.9	60.7–61.1	0.1	0.1–0.1
33	170.8	170.0–171.6	19.8	19.0–20.5	1.2	130.7	160.5	195.1	304.9	49.6	49.4–49.8	0.1	0.1–0.1
34	136.6	135.8–137.4	19.8	19.0–20.5	1.2	1.2	145.1	185.2	283.8	60.9	60.7–61.1	0.1	0.1–0.1

Note: 95%CI. 95% confidence interval; P5-P95. percentile 5 to 95 of iodine intake distribution; Ins. (%). prevalence of insufficient intake (<160 mcg); Exc. (%). prevalence of excessive intake (>600 mcg).

**Table 4:** Parameters of usual iodine intake estimates and Akaike Information Criterion according to Models, Multicenter Study of Iodine Deficiency, Brazil, 2023.

Models	S^2^b	95% CI Sb	S^2^w	95% CI S^2^w	S^2^	S^2^w/S^2^b	Lambda	AIC	Skewness	CV
Baseline	3.6	1.2–6.0	10.6	4.9–16.4	14.2	3.0	0.4	30,317.3	2.2	6.3
1	3.6	1.2–6.0	10.6	4.9–16.3	14.2	3.0	0.4	30,319.2	2.8	34.9
2	3.5	2.4–4.7	9.8	8.4–11.2	13.3	2.8	0.4	30,319.8	2.8	35.2
3	3.5	2.3–4.7	9.8	8.2–11.3	13.3	2.8	0.4	28,204.6	1.7	33.4
4	3.5	2.3–4.7	9.7	8.2–11.2	13.2	2.8	0.4	28,178.6	1.7	33.3
5	3.5	2.3–4.7	9.7	8.3–11.2	13.2	2.8	0.4	28,196.3	1.7	33.4
6	3.9	2.6–5.3	9.2	7.6–10.8	13.2	2.3	0.4	21,897.9	−1.2	27.5
7	3.5	2.3–4.7	9.8	8.3–11.2	13.2	2.8	0.4	28,081.6	1.6	33.3
8	3.6	2.3–4.8	9.7	8.3–11.2	13.3	2.7	0.4	28,147.4	1.7	33.3
9	3.5	2.3–4.7	9.7	8.2–11.2	13.2	2.8	0.4	28,170.3	1.7	33.3
10	3.9	2.6–5.2	9.2	7.6–10.7	13.1	2.4	0.4	21,893.2	−1.3	27.5
11	3.5	2.3–4.7	9.7	8.2–11.2	13.2	2.8	0.4	28,055.5	1.6	33.3
12	3.6	2.4–4.8	9.7	8.3–11.1	13.3	2.7	0.4	28,121.3	1.7	33.3
13	3.9	2.6–5.2	9.1	7.5–10.7	12.9	2.4	0.4	21,895.2	−1.3	27.5
14	3.5	2.3–4.7	9.7	8.3–11.1	13.2	2.8	0.4	28,047.0	1.6	33.3
15	3.5	2.3–4.7	9.7	8.2–11.2	13.2	2.8	0.4	28,113.0	1.6	33.3
16	3.8	2.5–5.1	9.1	7.5–10.7	13.0	2.4	0.4	21,786.2	−1.3	27.4
17	3.9	2.6–5.2	9.1	7.5–10.7	13.0	2.3	0.4	21,882.2	−1.2	27.5
18	3.5	2.3–4.7	9.7	8.2–11.2	13.2	2.8	0.4	27,990.0	1.6	33.2
19	4.0	2.6–5.3	9.1	7.5–10.8	13.1	2.3	0.4	21,784.2	−1.3	27.4
20	3.9	2.6–5.2	9.2	7.6–10.7	13.1	2.3	0.4	21,880.2	−1.3	27.5
21	3.9	2.6–5.2	9.2	7.6–10.7	13.1	2.3	0.4	21,771.5	−1.3	27.4
22	3.5	2.3–4.7	9.7	8.2–11.2	13.2	2.8	0.4	27,998.7	1.6	33.3
23	3.9	2.6–5.2	9.1	7.5–10.7	13.0	2.3	0.4	21,899.9	−1.3	27.5
24	3.9	2.6–5.2	9.2	7.6–10.8	13.1	2.4	0.4	21,790.9	−1.3	27.4
25	3.9	2.6–5.2	9.1	7.5–10.7	13.1	2.3	0.4	21,886.8	−1.2	27.5
26	3.9	2.6–5.2	9.1	7.6–10.7	13.1	2.3	0.4	21,778.1	−1.3	27.4
27	3.5	2.3–4.7	9.7	8.2–11.2	13.2	2.8	0.4	28,073.1	1.6	33.3
28	3.5	2.3–4.7	9.7	8.3–11.2	13.3	2.8	0.4	28,139.0	1.7	33.3
29	3.5	2.3–4.7	9.7	8.2–11.3	13.2	2.8	0.4	28,016.1	1.6	33.3
30	3.9	2.6–5.2	9.2	7.7–10.8	13.2	2.3	0.4	21,788.9	−1.3	27.4
31	4.0	2.7–5.3	9.2	7.7–10.8	13.2	2.3	0.4	21,884.8	−1.2	27.5
32	4.0	2.6–5.3	9.2	7.6–10.9	13.2	2.3	0.4	21,776.1	−1.3	27.4
33	3.5	2.3–4.7	9.8	8.2–11.3	13.3	2.8	0.4	28,024.8	1.6	33.3
34	3.9	2.6–5.2	9.1	7.5–10.7	13.0	2.3	0.4	21,773.5	−1.3	27.4

Note: S^2^b. interindividual variability; 95%CI. 95% confidence interval; S^2^w. intraindividual variability. S^2^. total variance; S^2^w/S^2^b. ratio of intraindividual to interindividual variability; AIC. Akaike Information Criterium; CV. Coefficient of variation.

## Data Availability

Not applicable.
